# Criminal Abortion

**Published:** 1860-03

**Authors:** 


### Criminal Abortion.

It is a somewhat startling, and to the moralist must be a disheartening fact, revealed by the statistician, as well as by the experience and observation of the medical practitioner, that a crime, in itself one of the foulest, and against which in times past the severest penalties have been attached, should, at this moment, be one of the most frequent not only in the older portions of the civilized world, but here in our very midst, where it has been supposed, from the character of the population and of the institutions under which we live, a higher and more healthy moral tone must necessarily prevail. We refer to the felonious act by which a blow is aimed at the life of a human being, while yet in its fœtal stage of existence, either for the purpose of concealing dishonor, or for the monstrous and more unnatural one of escaping the cares and responsibilities of maternity—an act which, three hundred years ago, subjected those convicted of its committal, to all the penalties, civil and ecclesiastical, inflicted on murderers.

Our attention has been called to the subject at this time by a treatise on criminal abortion, from the pen of one of our most pains-taking and careful investigators, Dr. H. R. Storer, who, it will be remembered, was appointed at the session of the American Medical Association, in May, 1857, chairman of a Committee to investigate the whole subject and report thereupon, with a view to the suppression, if possible of this growing evil. This paper contains much interesting information, and if it do as much for poor humanity as might be fairly expected, from the ability and good intentions of the author, he will have much reason for pleasant reflection.

It is not our object, nor is this the place, to enter into any extended analysis of this painfully interesting statement, but we have been so much struck with some of the results therein arrived at, particularly as showing the alarming prevalence of the evil in question, and its bearing upon the census returns, that we have thought a brief allusion to the subject might not be inappropriate, as simply calling attention to what, as has been well said, "appeals, in its medical as well as moral bearings, alike to our patriotism and humanity."

The effect of this species of child murder, when practiced to any considerable extent, upon the population, in diminishing its annual ratio of increase, is at once obvious; but we confess we were not prepared for the results given by Dr. Storer. Here we have statistical tables taken from the most trustworthy sources, not only in Europe, but in many parts of our own country; and, from the latter, it would appear that the native population in our own State of Massachusetts, where it may be fairly assumed every possible natural advantage exists for its rapid increase, is stationary or actually diminishing; the general increase being due to the excess of births over deaths among those of foreign origin. Allowing for the influence of western emigration in producing such a result, this can hardly be made to account for what is plainly evident from the statistical returns. It appears from tables, carefully compiled from the fifteen published Registration Reports of the State of Massachusetts, that the ratio of births to the population in this State, both foreign and American combined, was, in 1850, 1 in 36; and in 1855, 1 in 34; a ratio much smaller, says our author, than that obtaining in most countries in Europe, where, from the greater difficulties in living, and the other conditions arising from a crowded population, we should naturally expect this ratio to be at least comparatively small. Such, however, does not seem to be the case, although we find that in nearly all European countries it is rapidly diminishing. Thus, during the last century, it lessened in Sweden by a fifth; in Prussia by a fourth; in Denmark and England by a third; and in France by one-half. So, in the city of Paris alone, from 1817 to 1831, the births averaged about 1 in 26·87 inhabitants; while from 1846 to 1851 the average was 1 in 32.

If we now refer to the statistical tables as showing the ratio of still births, including abortions, to living births, we find this to be steadily increasing, and that it is probably greater in the State of Massachusetts at this moment than in Europe. For, in 1855 we find it about 1 to 15·5, being, at the same time, in France, 1 in 24, and in Austria 1 in 49.

Again, if we take the ratio of fœtal deaths to the general mortality, we find this higher in New York and Massachusetts, than in the countries of the old world, and still on the increase; having risen in the city of New York from 1 to 37, in 1805, to 1 in 13, in 1855; and in Massachusetts from 1 to 13·3, in 1851, to 1 in 10·4, in 1855—the last being greater than the ratio in New York in 1856.

The comparison of the number of abortions and premature births, with still births at full time, seems to establish still further the truth of the deductions already made. Thus, in New York, in 1838, the ratio was about 1 in 10; this having become as high, in 1856 as 1 in 4; while in Massachusetts, from 1850 to 1855, we blush to say it, the proportion was even eight times greater, that is, about 1 to ·5. Allowing for errors, and for the less efficient registration elsewhere, we are still forced to the conclusion, by a comparison of the ratio of births to the population in Massachusetts with that of the other countries alluded to; of still births, including abortions, to living births; of fœtal deaths to the general mortality; and of abortions and premature births, to still births at full time, that criminal abortion prevails to a greater extent in this State than in those countries. "Few persons," says Dr. Storer, "could have believed possible the existence of such frightful statistics, the result towards which they tend, or the dread cause from which they spring."

With regard to the proposed remedies for this evil, we confess we have little confidence in mere legislative enactments. Much, it is true, may possibly be done towards its partial suppression, by stringent laws, promptly and efficiently enforced; but when it is considered that this crime is fast becoming, if it has not already become, "an established custom," not confined to the unfortunate, but resorted to by the married of all ranks and classes of society, for the purpose of ridding themselves of what they have learned to regard as a burden too heavy to be borne, we fear little can be accomplished by legislation. It is rather to the medical profession, and to those more immediately entrusted with the morals of the community, that we are chiefly to look for the true remedy. The physician may do much by warning his patients against the dangers and guilt of this awful crime, and using the "greater vigilance lest he become its innocent and unintentional abettor;" and the moralist may do more by the inculcation of those principles in the young, that shall lead them to regard with abhorrence such a violation of the positive laws of God, involving, as it does, the guilt of murder, and a total indifference to the most sacred privileges with which woman is endowed.—*Boston Med. & Surg. Jour.*

